# Impact of the COVID-19 Pandemic on Meal Gathering in China

**DOI:** 10.3390/ijerph192416698

**Published:** 2022-12-12

**Authors:** Qing Chang, Yiheng Shu, Wuyang Hu, Xiaolei Li, Ping Qing

**Affiliations:** 1 College of Economics and Management, Huazhong Agricultural University, Wuhan 430070, China; 2 Research Center of Food Economics, Nutrition and Health, Huazhong Agricultural University, Wuhan 430070, China; 3 School of Business, Jiangnan University, Wuxi 214122, China; 4 Department of Agricultural, Environmental, and Development Economics, The Ohio State University, Columbus, OH 43210, USA

**Keywords:** COVID-19, meal gathering, food consumption, China

## Abstract

During the COVID-19 pandemic, the Chinese government adopted a series of preventative measures to control the spread of the virus. This paper studies the impact of the COVID-19 pandemic and its associated prevention methods on meal sharing in China. Meal gathering during multiple periods before and after the outbreak of COVID-19 is captured through two waves of online survey across China between March and June 2020, collecting a total of 1847 observations. We employ the difference-in-difference (DID) method to identify the causal effects of COVID-19 severity on meal sharing. The results show that relative to the same period in 2019, the frequency of meal gathering decreased sharply after the initial outbreak of the coronavirus in 2020 in both epicenters and non-epicenters. Furthermore, the impact of COVID-19 differed across different types of meal sharing. Our findings have implications for consumers, food service operators, as well as policymakers to understand the social and community impact of the pandemic and to adjust their coping strategies.

## 1. Introduction

As countries try to contain the spread of the highly infectious novel coronavirus (COVID-19), many preventative measures including “stay at home” orders and occasional lockdowns were adopted asking people to work together to reduce the impact of the virus by altering their routine activities [1,2,3]. Wuhan, a big metropolitan in central China, was among the first making the sacrifice to close to stop the pandemic. The rest of the country quickly followed suit and remained closed for several weeks. In addition to temporary lockdowns, people were asked to practice social distancing, which is described as reducing or avoiding non-essential gatherings, and substitute dine-in meals with carry-out meals. The impacts of the pandemic and coping methods are deep felt in many dimensions of social and private lives, including the food system.

As an important social activity in the Chinese society, meal gatherings for family, friends, and business purposes face direct threat from the ongoing COVID-19 pandemic. On the one hand, limited social events prevent people from engaging in meal gathering in indoor public spaces as indoor meal gathering and close physical contact facilitate easy and rapid spread of this pathogen [4]. Even the frequency and size of such gatherings at home have been reduced [5]. On the other hand, the highly contagious and deadly nature of the virus would further deter people from participating in communal meal gatherings, even without any social distancing recommendations. How has the COVID-19 pandemic affected Chinese people’s altitudes on social meal gatherings? Will they change their meal gathering behavior after the pandemic eases? Answering these questions will help assess the long-term economic and social impact brought about by this global health emergency.

Given the possibility that indoor gathering behavior may be affected by the ongoing pandemic, the goal of this study is to examine the impact of COVID-19 pandemic on food consumption behavior at the consumer level, with attention on meal gathering activities in China. Specifically, we aim to explore the changes of different types of meal gathering activities since the onset of the COVID-19 pandemic in different areas across China. Our first hypothesis is that the pandemic would negatively affect meal gatherings. Secondly, we look at geographic differences of the impact and hypothesize this to have a negative impact on meal gathering behavior in both epicenters and non-epicenters of the pandemic. Additionally, we hypothesize the impact to differ across different types of meal gatherings and would persist even after the pandemic eases.

Using a nationwide survey, this study contributes to the existing literature in the following two main ways: First, we investigate the changes in food consumption behavior among Chinese consumers, particularly meal gathering under the pandemic. This complements the current research on how a major public health event, such as the COVID-19 pandemic, can influence behavior of food consumption. We also provide some suggestions for retailers and policy makers to understand the impacts of the pandemic and adjust their operations. Second, by combining data collected through two waves of online surveys with the unique COVID-19 index, we are able to provide a more dynamic view on the impact of the pandemic on meal gathering.

The remainder of this paper develops as follows: Section 2 highlights the current literature on food consumption behavior in general as well as the influence on such behavior under the COVID-19 pandemic. Section 3 contains a detailed description of the methodology and data. Main regression results are displayed in Section 4. Section 5 offers discussion of the main findings, limitations and future research opportunities, and Section 6 concludes the analysis and offers policy implications.

## 2. Literature Review

The behavior of food consumption is influenced by cultural and social aspects [6]. Numerous past studies have highlighted the importance of understanding the cultural and ethical factors regarding food demand [7,8,9,10,11]. Chinese culture often attaches ceremonial purposes to meals and communal food sharing is seen across the country as a means to bond, entertain, make social arrangements, and even resolve disputes [12,13]. In addition to holiday occasions, meal gathering for business purposes is particularly common in China as a gesture to show sincerity and usually involves overmuch amount of food and drinks [13].

Since the onset of the pandemic, there have been dramatic changes in people’s food consumption behavior [14,15]. When considering the effect from a public health crisis, existing studies have focused primarily on consumer food purchase quantities as well as their nutrition compositions under COVID-19 [16,17,18]. Some studies have found that people increased the amount of food purchased and tended to consume more carbohydrates and high-energy foods during COVID-19 [6,19,20]. However, meal gatherings, as an important component of the food culture likely affected by a public health emergency, has not been well understood. Findings of the above-mentioned studies generally suggests that the COVID-19 pandemic brings significant behavior changes in terms of food consumption with more food consumed at home, therefore, one could argue that on the other end of the spectrum, meal gathering activities would also be affected, yet the current literature is lacking investigation into this segment of food consumption.

When considering the potential of long-lasting impact of the COVID-19 pandemic, existing studies show mixed findings on whether the overall amount of food consumption would recover in the long-run. Ruan et al. (2021) argues that food consumption behavior changes would likely be short-lived [21]. At the same time, Deloitte’s “Global State of the Consumer Tracker” monthly survey shows that although dine-out activities will recover from its current level, it will be difficult to reach the pre-pandemic level as shown by respondent’s intended behavior [22]. However, there is no clear evidence of the correlation between meal gathering and COVID-19. Therefore, we conduct a nationwide online survey on Chinese respondents’ current and future food consumption. This study focuses on the impact of COVID-19 on the behavior of food consumption at the consumer level, particularly on its impact on consumer meal gathering behavior and whether the pandemic may bring long-lasting impact and change the established meal gathering tradition. We examine the meal gathering sector of the food economy that has been directly affected by COVID-19 and is highlighted by the pandemic’s evolvement in China.

## 3. Methodology and Material

### 3.1. Data Sources

The data used in this article are based on the combination of two waves of surveys conducted by the research team through an online survey platform as well as the COVID-19 severity index captured through the Internet.

The first wave of the survey was administered in March 2020 to a random sample of respondents nationwide, from which a total of 1046 responses were obtained (Survey response rate was proprietary information held by the survey company). In addition to key research questions related to this study, which will be described in the next section, the survey includes detailed personal and family information of the respondents. These allow us to resample the same respondents in our second wave of the survey.

We conducted the second wave survey in June 2020 when the COVID-19 pandemic had largely been contained in China, and we were able to obtain responses from 601 individuals from the first wave. To balance the number of samples in the two waves, we recruited 200 additional responses in the second wave. As a result, a total of 1847 observations were obtained in the two waves of survey combined.

Our total observations cover 31 of the 34 provinces, autonomous regions, municipalities, and special administrative regions in China. We sampled from 31 provinces based on the proportion of confirmed COVID-19 cases in each province in the first quarter of 2020 announced by the National Health Commission of China (http://www.nhc.gov.cn/xcs/yqtb/list_gzbd_24.shtml accessed on 19 October 2022). Within each province, pure random sampling was adopted. Among the total 1847 observations, 248 are from Hubei province, where the pandemic was the most severe at the time, and 152 from Guangdong province, where the severity of the pandemic was second to Hubei. In addition, 122, 97, and 89 observations are from Henan, Hunan, and Zhejiang provinces, respectively where the pandemic was also more severe than the rest of the country. The remaining 1139 observations are from the remaining provinces/regions.

Sorting through the collected data, we removed responses who failed attention check questions and retained 1790 valid observations, with an effective response rate of 97%. Finally, we paired the survey data with the COVID-19 severity index of each city in the corresponding time periods (20 January 2020–20 February 2020 and June 2020). We will explain more details of these variables in the next section.

### 3.2. Variable Measurement

#### 3.2.1. Meal Gathering

Meal gathering is the key dependent variable of this study. Due to the complexity of meal gathering including the locations (restaurants/home), companions (family/friend/business partner), and occasions (holiday/business) of the gathering, the impact of COVID-19 may differ. Therefore, we divide meal gatherings into four different common forms [6,23]: away from home with family members, away from home with business partners, away from home with friends, and at home with non-family members.

For each of these four forms, we asked respondents to recall the number of such gatherings they have participated in four time periods: in the first wave, we asked respondents about the number of meal gatherings during the 2019 Lunar New Year season (from end of January to end of February 2019) and the 2020 Lunar New Year season (from Mid-January to Mid-February 2020); in the second wave, we asked respondents the number of meal gatherings in June 2019 and June 2020. We chose the Lunar New Year because it is the most important holiday of the year in China, and the Lunar New Year 2020 occurred roughly around the same time when COVID-19 outbroke. We choose an average summer month of June in the second wave in order to assess the impact of the pandemic on meal gathering when the pandemic in China began to subdue.

#### 3.2.2. COVID-19 Severity

In recent years, big data has become a popular way for researchers to acquire multidimensional information including health, food supply [24]. It has entered the public health field with their aptitude for real-time and dynamic feedback [25]. In the United States, researchers have successfully predicted flu outbreaks by analyzing Google Trends and accurately estimated flu levels one to two weeks earlier than reports published by the Centers for Disease Control and Prevention [26]. We utilize the big data method to mimic the dynamic evolution of the pandemic in China by incorporating the COVID-19 search index as a measure for the severity of the pandemic.

Since the evolvement of the pandemic has been highly fluid, we introduced a COVID-19 severity measure ‘COVID-19 search index’ which utilizes online data extraction methods to obtain the number of COVID-19 related searches on Baidu.com, the most popular search engine in China. This could help mitigate an inherent shortcoming of survey methods for not being able to respond to real-time changes. In this study, we developed a Baidu search index for COVID-19 pandemic information as a means to represent COVID-19 severity. Most of COVID-19 related web searches originating from China are recorded through the Baidu search engine http://www.baidu.com (accessed on 19 October 2022) [27]. As a result, we utilized the method of Internet crawling (also known as the web-crawler) to obtain the daily number of COVID-19 pandemic related searches in each Chinese city from the Baidu search engine. The search information was recorded from 20 January 2020 to 30 June 2020, to match the period of our survey data. We then obtained the COVID-19 pandemic search index by adding up the daily number of searches of each month for each city presented in our data.

#### 3.2.3. Other Control Variables and Sample Characteristics

We control demographic variables that may affect meal gathering. The series of control variables involved are all from the questionnaire. The coding, descriptions and descriptive statistics of the explanatory variables are shown in Table 1. From the perspective of individual characteristics, males accounted for 45.6% of the 1790 observations; the average age was 32.476; the average education level was 15.5 years; the average health level was 3.914 on a self-reported Likert scale from 1 to 5 corresponding to very poor to very good, respectively. An average household had about 3.4 members and the average household income in 2019 was ¥134,000 (CNY 1 ≈ USD 0.15), which is 5.127 as show in Table 1 after logarithmizing.

### 3.3. Model

To further investigate how meal gathering was affected by the pandemic, a two-period panel was constructed based on the two waves of surveys and several functional specifications were constructed.

Following Qian (2008) and Špička (2018), we adopt the difference-in-difference regression method [28,29]. We are able to construct a two-stage unbalanced panel data to analyze changes in meal gathering under different stages of the pandemic. The difference-in-difference model is as follows (We omit the notation differentiating the four types of meal gatherings since they all share the same model specification. Furthermore, instead of a discrete model corresponding to counts [30], we treat our data as continuous):

(1) $$Counts_{jimt}=\beta_{0}+\beta_{1}\mathrm{COVID}-19_{jtm}\times Year_{t}+\beta_{3}X_{it}+\tau_{t}+\theta_{j}+\varepsilon_{jimt}$$

Subscripts  j,i, m and t  represent cities, respondents, periods (January–February and June) and year, respectively. The dependent variable $Counts_{jimt}$ represents the number of household meal gatherings (away from home with family members, away from home with business partners, away from home with friends, and at home with non-family members); $\mathrm{COVID}-19_{jtm}$ represents the logarithmic value of the Baidu search index for information about COVID-19 severity. $Year_{t}$ takes a value of 1 for 2020, otherwise a value of 0. $X_{it}$ represents a series of control variables, including the respondents’ gender, age, age squared, years of education, health condition, household size and household income. $\theta_{j}\;$ representsan city fixed effect; $\tau_{t}$ is the time fixed effect; and $\varepsilon_{jitm}$ is the perturbation. Coefficient $\beta_{1}$ of the cross-multiplication term reflecting the average impact of the COVID-19 severity on household meal gathering.

## 4. Results

### 4.1. Summary Statistics

#### 4.1.1. Meal Gathering: Comparison between 2020 and 2019

**Figure 1:**
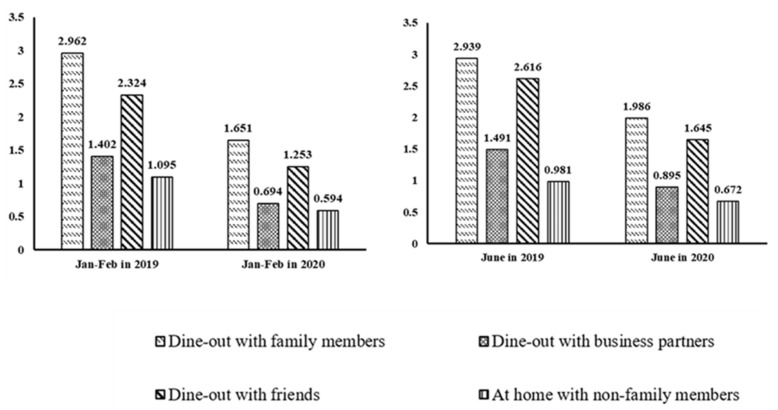
Meal gatherings in 2019 and 2020. Data are obtained through survey. Figures are plotted by Stata16.1.

We discuss the changes in meal gathering patterns over time (i.e., 2019 vs. 2020). Figure 1 and Table 2 break down the frequency of four types of meal gatherings over time. Figure 1 reports the sample mean of meal gatherings with different types during the two periods. Figure 1 shows that the number of meal gatherings during the outbreak period are all less than the same period in the previous year (i.e., each column represents a different type in Figure 1). This declining trend was supported by the results in Table 2 (Panel A). Especially for the January to February period, compared with 2019, the number of meal gatherings affected by the pandemic on all occasions dropped by at least 44.26%. The number of away from home meal gatherings with family members decreased the most in magnitude by 1.311 meals but at the lowest percentage change (44.26%); the largest percentage decrease occurred for gatherings away from home with business partners with a 50.5% drop. The number of at home meal gatherings with non-family members dropped the smallest in magnitude by 0.501 compared to 2019.

Figure 1 shows that the number of meal gatherings in June 2020 was also generally on the decline compared to that in the same period of 2019 but the magnitude of decline was smaller compared to early months in the year. Consistent with the conclusion in Figure 1, Table 2 (Panel B) reports that the number of all types of meal gatherings decreased during the outbreak period. The frequency of away from home gatherings with family members decreased by 0.953, a decrease of 32.43% compared with the same period last year. During the January-February period, the drop was 44.26%. The number of dine-outs with business partners decreased 39.97% compared with the same period in the previous year with a 50.5% decline observed during the January-February period. The number of away from home gatherings with friends in June 2020 decreased by an average of 37.12% compared to June 2019 and is also lower than the drop occurred during the January-February period. Finally, the frequency of meal gatherings at home with non-family members decreased by 31.50% compared with 2019, lower than the 45.75% reduction observed during the January–February period.

#### 4.1.2. Meal Gathering: Comparison between Epicenter and Non-Epicenter

In order to reflect the changes in meal gatherings along with the pattern of the pandemic, we also discuss the changes in meal gathering patterns over space (i.e., epicenters of the pandemic versus other regions). Table 3 shows the frequency of changes in meal gatherings among epicenters and non-epicenters. We use the data released by the National Health Commission of the People’s Republic of China and refer to the top five provinces with the highest number of confirmed cases as the epicenter, and the remaining provinces as non-epicenters. Judging from the four different types, meal gatherings were higher in June than in the January–February period for both epicenters and non-epicenters. In addition, no matter whether in January–February 2020 or in June 2020, most differences between epicenters and non-epicenters were insignificant. This result provides support for the second hypothesis that meal gatherings would be affected in both epicenters and non-epicenters. Interestingly, in the January–February period, the frequency of meal gatherings away from home with business partners and friends were both higher in epicenters than in non-epicenters.

### 4.2. Regression Results

Table 4 and Table 5 show regression results on the four different types of meal gatherings for the January-February period and June, respectively. The specific estimation results in Table 4 can be summarized as follows:

(1) After controlling for the provincial fixed effects and time fixed effects, COVID-19 has a significant negative impact on all four types of meal gatherings at the 1% significance level. This result conforms to our first hypothesis that the pandemic could negatively affect meal gatherings.
(2) Among the four types of meal gatherings, compared to the same period in 2019, between January and February in 2020, away from home gatherings with family members saw the most significant negative impact from COVID-19 with a reduction of 23.2% attributed to the pandemic, followed by away from home gatherings with friends (reduction of 18.5%), away from home gatherings with business partners (12.8% reduction), and lastly at home gatherings with non-family members (8.7% reduction).
(3) As for demographic variables, male consumers tended to have more meal gatherings away from home with family than female consumers. Consumers’ age had nonlinear impact on meal gatherings for all four categories. Higher education was associated with more away from home gatherings with business partners and friends. Healthier consumers would have more away from home gatherings with family. Larger household size and family income had positive association with more meal gatherings except that income was only significant for meals gatherings away from home with family or at home with non-family.

Table 5 shows that even though COVID-19 was mostly contained, it still had negative impact on meal gathering. Compared with the number of meal gatherings in the same period in 2019, the number of away from home gatherings with family members in June 2020 was down by 18.8% due to the impact of the pandemic, while the number of away from home gatherings with business partners fell by 11.6%; the number of away from home gatherings with friends dropped by 18.5%; and the number of at home gatherings with non-family members decreased by 5.7%. All decreases are statistically significant at the 1% level. Other variables had qualitatively similar impact as in Table 5. It is thus clear by comparing Table 4 and Table 5 that the negative impact of COVID-19 on meal gatherings had reduced between the January–February period and June, but the significant negative impact still lingered. The result is consistent with the last hypothesis that the impact of the pandemic can differ across different types of meal gatherings and could persist even after the pandemic eases.

## 5. Discussion

Relying on a survey in China, this study aims to analyze the impact of COVID-19 pandemic on food consumption at the consumer level, with a particular focus on meal gatherings. We find that the COVID-19 pandemic has likely decreased the frequency of meal gatherings and changed consumer behavior toward meal gatherings. When the pandemic was at its initial stage (i.e., between January and February 2020), all four types of meal gatherings saw a significant decrease in number. However, with the temporary remission of the pandemic (e.g., June 2020), the impact of the COVID-19 pandemic appears to have diminished although not completely compared to the same period in the previous year. The main reason for this observation is that households generally reduced dining out during the pandemic. In addition, during the Spring Festival, people often returned to their hometowns from other regions and gathered with family or friends more frequently. In June 2020, the initial spread of the virus in China was under control. In-person meetings and business activities began to resume in many parts of China, and many preventative measures were lifted. However, with the increasing awareness of the high infectivity and high treatment cost of the disease [19], the number of communal meal sharing is still at a low level. Consistent with previous studies [6,31], we also find that the COVID-19 crisis changes the behavior of meal gathering across the country, which suggests that the impact of the COVID-19 pandemic is more general but not region specific.

In addition, we also note several limitations of our study and some useful topics for future research. First, as an online survey utilizing random sampling with the actual response rate being unavailable to us, we cannot ensure the representativeness of our sample. Biases related to survey nonresponse and self-selection could be present. It would be worthwhile for future studies to adopt alternative sampling strategies such as using a probability-based sampling technique although such a method may incur significantly higher cost. Second, our COVID-19 search index can only capture online searches via Baidu.com. Although Baidu.com has the largest market share, it only accounts for around 59% of the Chinese search engine market (Data sources: Search Engine Market Share China, https://gs.statcounter.com/search-engine-market-share/all/china accessed on 19 October 2022). Future research could consider including searches from more platforms to make the severity measure more representative. Third, our attempts to explore the long-term impact of the pandemic on food consumption/meal gatherings was limited to survey responses from two surveys in 2019 and 2020. Respondents could experience some level of recall bias. Similarly, our results are also limited by the timeliness of our data. Although we believe our findings properly capture respondents’ behavior during the time of the study, since the initial outbreak of the pandemic was about three years ago, it would be useful to revisit the issues and assess the pandemic’s long-term impact on food consumption.

## 6. Conclusions and Policy Implications

### 6.1. Conclusions

Using two waves of a national survey and the dynamic data extracted from COVID-19 related online searches, we assess how the COVID-19 pandemic affected Chinese consumers’ meal gathering. Several findings emerge from our analysis.

First, compared to 2019, COVID-19 significantly reduced the frequency of meal gatherings in 2020. As expected, the impact was less pronounced in June when the pandemic was largely contained compared to early 2020 when the disease first outbroke. Second, the impact of COVID-19 differed across different types of meal gatherings. For the four types of meal gatherings we considered, COVID-19 had the most significant negative impact on gatherings away from home with family, followed by dine-outs with friends, dine-outs with business partners, and then at home with non-family members. Third, in terms of spatial difference, meal gatherings in both epicenters and non-epicenters became less frequent, but they exhibited statistically similar changes. This indicates that the COVID-19 crisis triggers nationwide changes in meal sharing behavior, the effects of which are observed beyond epicenters.

### 6.2. Policy Recommendations and Implications

By reflecting on our findings from this study and the ongoing COVID-19 pandemic, we propose several recommendations from a food retailer’s perspective and from a policymaker’s perspective. Our study shows that meal gathering frequencies were negatively impacted by the COVID-19 pandemic. This holds important implications for Chinese retail food businesses that should consider adopting more flexible forms of sales and rethink their marketing strategies during uncertain times. On the one hand, food retailers including restaurants should consider adjusting their operation methods, such as enhancing online deliveries and streamlining the variety of items offered to ease the financial burden on restaurants suffering from reduced meal sharing customers. On the other hand, food retailers should consider preventive measures in order to build trust among potential customers and eventually encourage patrons back to restaurants for meal sharing. Since communal meal sharing will still be an integral part of the Chinese tradition, policies targeting at mitigating disease transmissibility such as mandating the use of serving utensils for meal sharing should be considered. Catering business practitioners could consider dividing shared dishes into individual serving dishes for communal meal sharing. These measures could encourage food retailers to shift from a way of communal meal sharing that can often be associated with easy transmission of infectious diseases to a healthier option to curb uncertain health emergency events such as the COVID-19 pandemic.

From a society point of view, our use of the internet search index as a proxy for the severity of the COVID-19 pandemic is shown associated with household food consumption behavior. As the internet is becoming an essential part of people’s daily life, one should not overlook the power of information on the internet and its ability to guide consumption behavior. We recommend policy makers to consider utilizing the internet as a tool to facilitate socially desirable outcomes and create a more resilient food system. This includes making cautionary notes to limit the spread of false information, providing more transparent and source-verifiable information, and disseminating knowledge on food safety and healthful eating habits.

## Figures and Tables

**Table 1:** Variable and descriptive statistics.

Variable	Time	Mean	Std. Dev.	Min	Max
Panel A: Jan-Feb from survey wave one (N = 1006)					
COVID-19 (search index)	2020	5.502	0.349	4.493	6.226
Age	2019	31.334	7.742	15	66
	2020	32.334	7.742	16	67
Age^2^	2019	1041.704	547.114	225	4356
	2020	1105.372	562.365	256	4489
Male	2020	0.448	0.498	0	1
Education (in years)	2020	15.545	1.483	6	19
Healthy (1 very poor; 5 very healthy)	2020	3.932	0.692	2	5
Household-size	2020	3.461	1.059	1	10
Log (income) (pre-tax)	2019	5.147	0.348	3.699	5.778
	2020	5.047	0.379	3.398	5.799
Panel B: June from survey wave two (N = 784)					
COVID-19	2020	4.953	0.284	4.035	5.659
Age	2019	31.658	7.472	16	66
	2020	32.658	7.472	17	67
Age^2^	2019	1057.992	525.372	256	4356
	2020	1122.309	540.119	289	4489
Male	2020	0.466	0.499	0	1
Education	2020	15.548	1.498	6	19
Healthy	2020	3.890	0.719	1	5
Household-size	2020	3.305	0.943	1	8
Log (income)	2019	5.164	0.320	3.699	5.778
	2020	5.111	0.343	3.477	5.778
Panel C: Meal gathering from two waves of surveys combined (N = 1790)					
COVID-19	2020	5.262	0.422	4.035	6.226
Male	2020	0.456	0.498	0	1
Age	2020	32.476	7.625	16	67
Age^2^	2020	1112.790	552.643	256	4489
Education	2020	15.546	1.489	6	19
Healthy	2020	3.914	0.704	1	5
Household-size	2020	3.393	1.013	1	10
Log (income)	2020	5.127	0.342	3.574	5.789

Data are obtained through survey. Results are calculated by Stata16.1.

**Table 2:** Frequencies of meal gathering.

	(1) Number of Times per Month in 2019 ^#^	(2) Number of Times per Month in 2020 ^#^	(3) Difference (2019–2020) ^^^	(4) Percent Decrease from 2019 to 2020
Panel A: Jan–Feb				
Dine-out with family	2.962 (3.818)	1.651 (2.618)	1.311 *** (0.000)	44.26%
Dine-out with business	1.402 (3.031)	0.694 (1.432)	0.708 *** (0.000)	50.50%
Dine-out with friends	2.324 (3.356)	1.253 (1.748)	1.071 *** (0.000)	46.08%
At home with non-family	1.095 (1.854)	0.594 (1.202)	0.501 *** (0.000)	45.75%
Panel B: June				
Dine-out with family	2.939 (3.710)	1.986 (2.790)	0.953 *** (0.000)	32.43%
Dine-out with business	1.491 (2.573)	0.895 (1.959)	0.596 *** (0.000)	39.97%
Dine-out with friends	2.616 (2.946)	1.645 (2.031)	0.971 *** (0.000)	37.12%
At home with non-family	0.981 (1.832)	0.672 (1.501)	0.309 *** (0.000)	31.50%

Data are obtained through survey. **^#^** Numbers in parentheses are standard deviations. **^^^** Numbers in parentheses are *p*-values of the corresponding *t*-test for differences. *** indicates significance at the 1% level. Results are calculated by Stata16.1.

**Table 3:** Meal Gatherings in Epicenters and Non-Epicenters of the Pandemic (Frequencies).

	(1) Epicenter ^#^	(2) Non-Epicenter ^#^	(3) Difference (Epicenter–Non-Epicenter) ^^^
Panel A: 2020 (Jan–Feb)			
Dine-out with family	1.698 (2.443)	1.631 (2.631)	0.067 (0.706)
Dine-out with business	0.816 (1.769)	0.641 (1.254)	0.176 * (0.073)
Dine-out with friends	1.439 (2.037)	1.173 (1.602)	0.267 ** (0.026)
At home with non-family	0.580 (1.113)	0.601 (1.240)	−0.021 (0.806)
Panel B: 2020 (June)			
Dine-out with family	2.103 (3.206)	1.913 (2.496)	0.190 (0.354)
Dine-out with business	0.850 (1.417)	0.923 (2.232)	−0.073 (0.613)
Dine-out with friends	1.757 (2.228)	1.576 (1.897)	0.182 (0.223)
At home with non-family	0.744 (1.416)	0.627 (1.552)	0.117 (0.289)

Data are obtained through survey. **^#^** Numbers in parentheses are standard deviations. **^^^** Numbers in parentheses are *p*-values of the corresponding *t*-test for differences. **, and * indicate significance at the 5% and 10% levels, respectively. Results are calculated by Stata16.1.

**Table 4:** The impact of the pandemic on the number of meal gatherings from January to February in 2019 and 2020.

Variables	(1)	(2)	(3)	(4)
	Dine-Out with Family	Dine-Out with Business	Dine-Out with Friends	At Home with Non-Family
COVID-19	−0.232 ***	−0.128 ***	−0.185 ***	−0.087 ***
	(0.026)	(0.019)	(0.021)	(0.012)
Male	0.488 ***	0.101	0.123	0.013
	(0.158)	(0.109)	(0.121)	(0.070)
Age	0.053	0.084 **	0.078 *	0.033
	(0.053)	(0.034)	(0.040)	(0.024)
Age^2^	−0.001 *	−0.001 ***	−0.001 ***	−0.001 **
	(0.001)	(0.000)	(0.001)	(0.000)
Education	0.010	0.079 **	0.071 *	0.023
	(0.057)	(0.035)	(0.040)	(0.025)
Healthy	0.325 ***	0.084	0.041	0.056
	(0.089)	(0.059)	(0.082)	(0.046)
Household-size	0.140 **	0.189 ***	0.178 **	0.179 ***
	(0.071)	(0.071)	(0.076)	(0.034)
Log (income)	0.306 *	0.002	0.290	0.216 **
	(0.180)	(0.147)	(0.192)	(0.098)
Constant	−1.021	−2.226 **	−1.954	−1.670 **
	(1.553)	(1.045)	(1.295)	(0.760)
City fixed effects	Y	Y	Y	Y
Time fixed effects	Y	Y	Y	Y
Observations	2012	2012	2012	2012
Adjusted R^2^	0.058	0.040	0.057	0.053

Robust standard errors in parentheses. ***, **, and * indicate significance at the 1%, 5% and 10% levels, respectively. Results are calculated by Stata16.1.

**Table 5:** The impact of the pandemic on the number of meal gatherings in June 2019 and June 2020.

Variables	(1)	(2)	(3)	(4)
	Dine-Out with Family	Dine-Out with Business	Dine-Out with Friends	At Home with Non-Family
COVID-19	−0.188 ***	−0.116 ***	−0.185 ***	−0.057 ***
	(0.033)	(0.023)	(0.026)	(0.017)
Male	0.279 *	0.300 **	0.313 **	0.106
	(0.165)	(0.120)	(0.129)	(0.085)
Age	0.017	0.118 ***	0.030	−0.013
	(0.065)	(0.037)	(0.047)	(0.030)
Age^2^	−2.58 × 10^−4^	−0.002 ***	−0.001	−1.82 × 10^−5^
	(0.001)	(0.000)	(0.001)	(0.000)
Education	−0.038	−0.016	0.045	−0.030
	(0.054)	(0.031)	(0.037)	(0.022)
Healthy	0.339 ***	0.232 ***	0.237 ***	0.220 ***
	(0.107)	(0.088)	(0.088)	(0.065)
Household-size	0.166 *	0.169 ***	0.151 *	0.241 ***
	(0.091)	(0.061)	(0.088)	(0.051)
Log (income)	0.394	0.287	0.426 *	0.167
	(0.291)	(0.216)	(0.221)	(0.171)
Constant	−1.003	−3.081 **	−1.109	−0.380
	(1.742)	(1.410)	(1.619)	(1.212)
City fixed effects	Y	Y	Y	Y
Time fixed effects	Y	Y	Y	Y
Observations	1568	1568	1568	1568
Adjusted R^2^	0.066	0.058	0.077	0.045

Robust standard errors in parentheses. ***, **, and * indicate significance at the 1%, 5% and 10% levels, respectively. Results are calculated by Stata16.1.

## Data Availability

The data used to support the findings of this study can be made available by the corresponding author upon request.

## References

[1] Fang H.M., Wang L., Yang Y. (2020). Human Mobility Restrictions and The Spread of The Novel Coronavirus (2019-nCoV) in China. J. Public Econ..

[2] Rutz C., Loretto M.C., Bates A.E., Davidson S.C., Cagnacci F. (2020). COVID-19 Lockdown Allows Researchers to Quantify The Effects of Human Activity on Wildlife. Nat. Ecol. Evol..

[3] Gruère G., Brooks J. (2020). Viewpoint: Characterising Early Agricultural and Food Policy Responses to The Outbreak of COVID-19. Food Policy.

[4] Quadri S.A., Padala P.R. (2021). An aspect of Kumbh Mela massive gathering and COVID-19. Curr. Trop. Med. Rep..

[5] Malone T., Schaefer K.A., Lusk J.L. (2021). Unscrambling U.S. Egg Supply Chains Amid COVID-19. Food Policy.

[6] Huber B.C., Steffen J., Schlichtiger J., Brunner S. (2021). Altered nutrition behavior during COVID-19 pandemic lockdown in young adults. Eur. J. Nutr..

[7] Drury R. (2011). Hungry for Success: Urban Consumer Demand for Wild Animal Products in Vietnam. Conserv. Soc..

[8] Davidson K., Pan M., Hu W., Poerwanto D. (2012). Consumers’ Willingness to Pay for Aquaculture Fish Products vs Wild-Caught Seafood—A Case Study in Hawaii. Aquac. Econ. Manag..

[9] Soley G., Hu W., Vassalos M. (2019). Willingness to Pay for Shrimp with Homegrown by Heroes, Community-Supported Fishery, Best Aquaculture Practices, or Local Attributes. J. Agric. Appl. Econ..

[10] Li R., Lee C., Lin Y., Liu C. (2020). Chinese consumers’ willingness to pay for organic foods: A conceptual review. Int. Food Agribus. Manag. Rev..

[11] Shi X., Zhang X., Xiao L., Li B., Lu Z. (2020). Public Perception of Wildlife Consumption and Trade During The COVID-19 Outbreak. Biodivers. Sci..

[12] Newman J.M. (2004). Food Culture in China.

[13] Ma G. (2015). Food, Eating Behavior, and Culture in Chinese Society. J. Ethn. Foods.

[14] Chenarides L., Carola G., Jayson L.L., Iryna P. (2021). Food Consumption Behavior during the COVID-19 Pandemic. Agribusiness.

[15] Filimonau V., Le H.V., Sean B., Vladimir A.E. (2021). The COVID-19 Pandemic and Food Consumption at Home and Away: An Exploratory Study of English Households. Socio-Econ. Plan. Sci..

[16] Mahajan K., Tomar S. (2021). COVID-19 and Supply Chain Disruption: Evidence from Food Markets in India. Am. J. Agric. Econ..

[17] Chang H.H., Meyerhoefer C.D. (2020). COVID-19 and The Demand for Online Food Shopping Services: Empirical Evidence from Taiwan. Am. J. Agric. Econ..

[18] Li X., Li J., Qing P., Hu W. (2021). COVID-19 and The Change in Lifestyle: Bodyweight, Time Allocation, and Food Choices. Int. J. Environ. Res. Public Health.

[19] Muresan I.C., Harun R., Brata A.M., Brata A.M., Brata V.D., Chiciudean D.I., Tirpe O.P., Porutiu A., Dumitras D.E. (2022). Factors Affecting Food Consumers’ Behavior during COVID-19 in Romania. Foods.

[20] Batlle-Bayer L., Aldaco R., Bala A., Puig R., Laso J., Margallo M., Vázquez-Rowe I., Antó J.M., Fullana-i-Palmer P. (2020). Environmental and nutritional impacts of dietary changes in Spain during the COVID-19 lockdown. Sci. Total Environ..

[21] Ruan J., Cai Q., Jin S. (2021). Impact of COVID-19 and Nationwide Lockdowns on Vegetable Prices: Evidence from Wholesale Markets in China. Am. J. Agric. Econ..

[22] Deloitte (2022). State of the Consumer in the COVID World. https://www2.deloitte.com/global/en/pages/consumer-business/articles/deloitte-state-of-the-consumer-tracker.html.

[23] Oh A., Erinosho T., Dunton G., Perna F.M., Berrigan D. (2014). Cross-sectional examination of physical and social contexts of episodes of eating and drinking in a national sample of US adults. Public Health Nutr..

[24] Bragazzi N.L., Dai H., Damiani G., Behzadifar M., Martini M., Wu J.H. (2020). How Big Data and Artificial Intelligence Can Help Better Manage The COVID-19 Pandemic. Int. J. Environ. Res. Public Health.

[25] Zhao C.H., Yang Y.H., Wu S.Y., Wu W.C., Xue H.T., An K., Zhen Q. (2020). Search Trends and Prediction of Human Brucellosis Using Baidu Index Data from 2011 to 2018 in China. Sci. Rep..

[26] Carneiro H.A., Mylonakis E. (2009). Google Trends: A Web-Based Tool for Real-Time Surveillance of Disease Outbreaks. Clin. Infect. Dis..

[27] Liu K., Wang T., Yang Z.C., Huang X.D., Milinovich G.J., Lu Y., Jing Q., Xia Y., Zhao Z., Yang Y. (2016). Using Baidu Search Index to Predict Dengue Outbreak in China. Sci. Rep..

[28] Qian N. (2008). Missing Women and The Price of Tea in China: The Effect of Sex-Specific Earnings on Sex Imbalance. Q. J. Econ..

[29] Špička J. (2018). How Does Public Investment Support Change the Capital Structure and Productivity of Small Enterprises? An Empirical Study of The Food Industry. Int. Food Agribus. Manag. Rev..

[30] Hu W., Cox L.J., Wright J., Harris T.R. (2008). Understanding Firms’ Relocation and Expansion Decisions Using Self-Reported Factor Importance Rating. Rev. Reg. Stud..

[31] Atsız O., Cifci I. (2021). Can we imagine the meal-sharing economy without service providers? The impact of COVID-19. J. Hosp. Tour. Manag..

